# Improving adherence to ante-retroviral treatment for people with harmful alcohol use in Kariobangi, Kenya through participatory research and action

**DOI:** 10.1186/1471-2458-12-677

**Published:** 2012-08-20

**Authors:** Caleb J Othieno, Obondo A, Mathai M

**Affiliations:** 1 Senior Lecturer, Department of Psychiatry, School of Medicine, College of Health Sciences, University of Nairobi, P.O. Box 19676 – 00202, Nairobi, Kenya; 2 Senior Lecturer, Psychiatry Social Work, Department of Psychiatry, School of Medicine, College of Health Sciences, University of Nairobi, P.O. Box 19676 – 00202, Nairobi, Kenya; 3 Lecturer, Department of Psychiatry, School of Medicine, College of Health Sciences, University of Nairobi, P.O. Box 19676 – 00202, Nairobi, Kenya

**Keywords:** Alcohol, PRA, ARV, PLWHA

## Abstract

**Background:**

Harmful alcohol use has been linked to the spread of HIV in Kenya. It also adversely affects those on antiretroviral (ARV) treatment through poor compliance. This study using participatory research and action (PRA) methods sought to understand factors related to alcohol abuse and non-adherence and to formulate appropriate interventions in a sample of people living with HIV and AIDS (PLWHA) who were also abusing alcohol, at Kariobangi in Nairobi, Kenya.

**Methods:**

Entry into the community was gained through previous PRA work in that community and PLWHA were recruited through snowballing. Working together with the community members, the researchers explored the participants’ understanding of alcohol use problem, its effects on compliance to ARV treatment and discussed possible action areas through PRA techniques that included focus group and market place discussions; visual aids such as spider diagrams, community mapping and ranking. Follow-up meetings were held to discuss the progress.

**Results:**

By the final meeting, 67 PLWHA and 19 community members had been recruited. Through discussions, misconceptions regarding alcohol use were identified. It emerged that alcohol abuse was poorly recognised among both the community and health workers. Screening for alcohol use was not routinely done and protocols for managing alcohol related disorders were not available at the local health centres providing ARVs. The study participants identified improving communication, psychoeducation and screening for alcohol use as possible action areas. Poverty was identified as a major problem but the interventions to mitigate this were not easy to implement.

**Conclusion:**

We propose that PRA could be useful in improving communication between the health workers and the clients attending primary health care (PHC) facilities and can be applied to strengthen involvement of support groups and community health workers in follow up and counselling. Integrating these features into primary health care (PHC) would be important not only to PLWHA but also to other diseases in the PHC setting . Longer term follow up is needed to determine the sustained impact of the interventions. Problems encountered in the PRA work included great expectations at all levels fostered by handouts from other donors and cognitive impairment that interfered with constructive engagement in some of the PLWHA.

## Background

There is a high prevalence of HIV infection in sub-Saharan Africa [1]. In Kenya, the majority of HIV infection is through heterosexual transmission [2]. In the report the Kenya National AIDS Council estimates that there are 1.4 million people living with HIV in Kenya. The adult HIV prevalence ranges from 0.81% in the North-Eastern provinces to 14.9% in Nyanza with higher prevalence rates ranging from 26% to 58% depending on the testing method used [3]. A range of socio-economic determinants, systems and resource constraints limit efforts to treat those infected and to reduce new infection [2]. The report noted that over two-thirds of resources are directed towards voluntary testing and counselling while no specific programmes target the PLWHA. It is therefore important to explore and implement programmes that can help to reduce the rate of new infections. One of the interventions that has been suggested is the reduction of harmful alcohol use.

**Figure 1:**
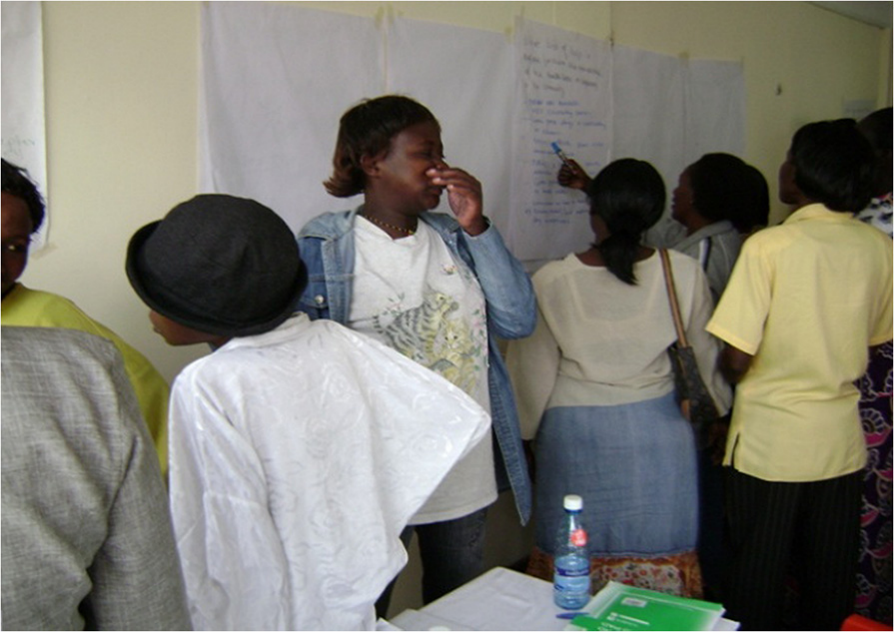
Participants engaged in "marketplace" discussions.

**Figure 2:**
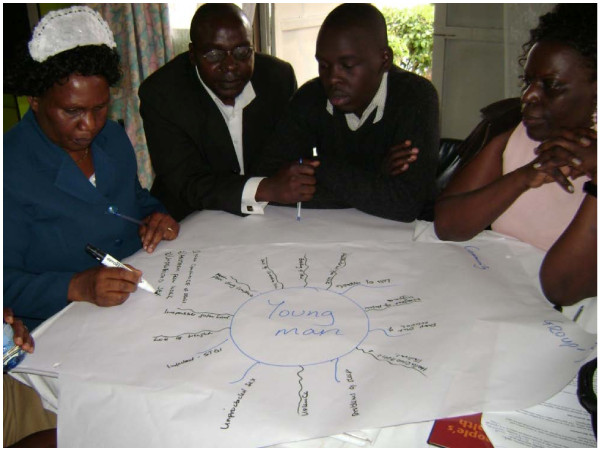
Use of a spider diagram to elicit the participants' feelings on the effects of alcohol on a young man.

**Figure 3:**
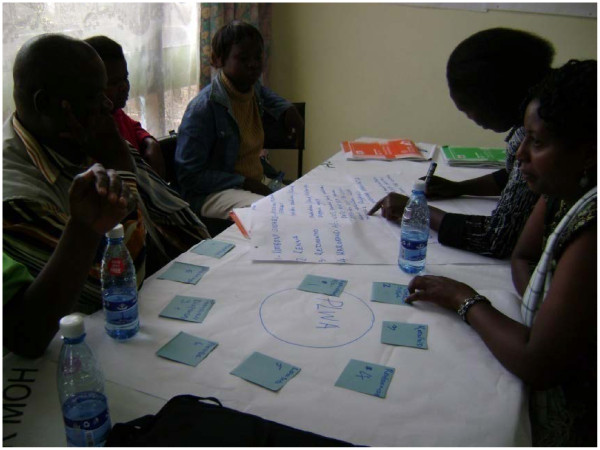
Participants using the Venn diagram to map out stakeholders working with PLWHA.

Harmful alcohol use is used to mean a pattern of use that is causing damage to health, either physical or mental [4]. It is a phenomenon that is widespread in the region. Earlier studies in Kenya showed that approximately 50% of the general population had harmful alcohol use [5,6]. Further studies indicated that although an estimated 70% of females and 45% of males in east and southern Africa (ESA) abstain from alcohol, the region had the highest consumption of alcohol per drinker globally [7]. The review further showed that the prevalence of harmful drinking in ESA was more common in those who consumed high quantities per session leading to high frequency of intoxication.

Alcohol use may affect HIV prevention and AIDS treatment in a number of ways. Alcohol use leads to disinhibition through its overall psychodepressant actions on the brain [8]. Although controversial it is thought that the disinhibition from alcohol intake may lead to an increased risk of unsafe sexual behaviours [9]. Alcohol use may undermine adherence to treatment [10,11], while alcohol interacts in a complex way with antiretrovirals (ARVs) leading to increased hepatotoxicity [12]. In theory therefore reduced harmful alcohol intake could lead to improved compliance and reduced risky sexual behaviour thus reducing the rates of new infections and slowing the progression of the epidemic.

The World Health Organisation (WHO) action plan for the prevention and control of non-communicable diseases [13] and the WHO report by the secretariat [14] proposed certain areas of focus that could be addressed by member states with regard to harmful alcohol use. These included the development of effective services for all people with alcohol-use disorders including those affected by HIV/AIDS. However little has been done in implementing this policy. The few services for alcohol detoxification and rehabilitation in Nairobi are privately run, with only one public centre based at Mathari Hospital. Thus there is a need to strengthen primary level services and to involve communities in identifying and reducing harmful alcohol use.

Strategies for reducing harmful alcohol use include physician advice, taxation, roadside random breath testing, restricted sales access and advertising bans. Chisolm et al. [15] reviewing cost effectiveness of these different strategies concluded that taxation would be less cost effective in populations with a low prevalence of heavy drinking. They noted that in Africa, since a substantial amount of alcohol consumed is produced and sold through illegal outlets, increasing taxation may actually increase the volume of illicit brew consumed. Offering advice in primary care centres and roadside breath testing were found to be the least cost effective in areas with a high prevalence of heavy drinkers (more than 5%) such as Europe or North America, but more suitable for populations with a concentration of fewer, but heavy drinkers, such as in Africa. A recent study in South Africa among a small group (n = 112) of South African female alcohol users showed that the women were responsive to behavioural interventions [16]. Cognitive behavioural therapy (CBT) has been shown to be effective in treating people with alcohol use problems in Kenya [17,18], however its use is still not widespread. Given that in most instances there are few or no effective interventions for managing people with harmful alcohol use and noting the adverse effects that alcohol has on those on ARVs we sought to explore the problem using participatory research and action (PRA) methods. Although PRA has been used in Africa in areas such as sociology and social psychology, education and agriculture its use in medicine and in particular psychiatry has been minimal. In Kenya PRA has been used in a health education, sanitation and water project [19].

### Objective

To explore the factors related to harmful alcohol use and identify interventions aimed at improving adherence to antiretroviral drugs among PLWHA who also use alcohol in a harmful way using participatory research and action (PRA) methods.

## Methods

### Ethical approval

We sought and obtained permission from the Kenyatta National Hospital’s ethical and research committee as well as the Mathari Hospital administration. Permission was also sought from the Ministry of Education Science and Technology to engage in the community work. Although the PRA process is a non-invasive procedure, confidential details needed to be recorded and therefore the participants were required to sign informed consent before enrolment into the project. They were assured that their individual identities would not be revealed in any reports or publications without their consent. The participants consented to being photographed at the meeting.

### Participants

Kariobangi area, in the eastern side of Nairobi City is densely populated, largely with semi-permanent houses and low income inhabitants. There are various support groups working with PLWHA which had participated in previous PRA work with members of the research team [20] The groups were: Women Fighting AIDS in Kenya (WOFAK), Kenya Widows and Orphans Support Group, Kenya Network of Women - Korogocho group, Maendeleo Afya Kwa Wote (MAKWAK), Rehema Day Care and Orphan Projects. In addition there was the group called “I am Worth Defending” and the Kariobangi Health Centre community based care for HIV and tuberculosis (TB).

## PRA methods

Participatory Research and Action (PRA) also known as Participatory Action Research (PAR) or Action research has been defined as a research approach that involves active participation of stakeholders, those whose lives are affected by the issue being studied, in all phases of research for the purpose of producing useful results to make positive changes [21]. It stems from the philosophy that human beings are capable of analysing and solving their problems. It is collaborative in nature and the researchers and participants identify the problem together. It involves learning from each other and understanding one another’s perspectives [22] Thus PRA discourages a top down approach in which a researcher or health worker comes with ready solutions. The PRA process goes through the three phases. The initial phase involves identifying issues to be addressed (in consultation with the study subjects). This is also known as the “listening phase”. This is followed by formulating the actions to be taken and the actual actions. In this study we worked with people living with HIV and AIDS to understand why they abuse alcohol leading to non-compliance and to try to identify the action areas.

Various methods are designed to facilitate the discussions and encourage participation during the PRA process as described by Rene [23] These can be adapted to suit particular situations. The ones used in this project were focus group discussions, brainstorming, role plays, market place discussions, spider diagrams and Venn diagrams. Additionally ranking and scoring and display using star charts helped the participants to identify priority areas.

The contact persons for the organisations described above together with the chief in Korogocho and Kariobangi communities formed the entry points to the community and helped to identify a few PLWHA who agreed to attend the initial meeting. Through snowballing additional PLWHA were recruited into the project. Additional recruitment was through the health workers at the local clinics who were requested to identify individuals who were on ARV treatment and were also using alcohol.

At the initial meeting, there were 10 PLWHA, 19 participants from the community and 5 health workers (community nurses from Mathari Hospital and mental health workers from Kariobangi Health Centre). The community participants included social workers, members of community based organisations (CBOs), family members, church leaders, and members of support and counselling groups. The authors facilitated the meeting.

At the first meeting the project leaders explained the purpose of the project and the participatory role each member was supposed to play. Through focus group discussions and the PRA tools the following issues were discussed: perceptions regarding alcohol use in the community, problems associated alcohol use and its effects on compliance to ARV. This was a “listening phase” of the programme.

In a second meeting the same participants (10 PLWHA, 19 participants from the community and 5 health workers) met again. Role plays and case vignettes were used to discuss and draw perceptions on the health problems associated with alcohol use. A “marketplace” approach was adopted to bring out issues of concern. In the market place discussions various issues thought to be important from previous discussions were put up on flip charts around a room. Each chart had a different theme. Participants moved around the room and mingled freely while discussing the issues (Figure 1). At each station there was a person who monitored and recorded the points that arose from the interactions. Before the meeting ended, the participants reflected and collectively analysed the causes and responses to problems identified. Specific persons were then assigned tasks for intervention.

Spider diagrams (Figure 2) refer to the figure obtained when a point for discussion is put at the centre on a flipchart and then as the participants discuss various ideas are connected to the central body. The strands connecting the ideas with the original theme at the centre form the legs of the spider. Spider diagrams were used to identify the stakeholders working with PLWHA and alcohol dependent individuals.

Venn diagrams (Figure 3) were used to map out and visualise the type and strength of relationship that stakeholders working with the PLWHA in the community have with each other. In this method the participants identify the various stakeholders and write the names on small pieces of paper. These are then laid down on the table. The distance, proximity and strength of the relationships are reflected in how the pieces of paper are laid out.

By the third meeting, held in October 2008, sixty seven PLWHA were now participating in the meeting, as support for the process grew.

A final review meeting was held in July 2009. At this meeting there were 81 PLWHA. Out of these only 41 had been present at the 3^rd^ meeting. Three health workers and two community based workers attended the meeting, with a total of 46 participants together with the 41 PLWHA.

## Results

The number of PLWHA that attended the meetings are shown in Table 1. The socio-demographic variables are summarised in Table 2. The majority of the PLWHA were socially disadvantaged, unemployed, and with low education. A large number were widowed or separated.

**Table 1:** Participatory Research and Action meetings

**Meeting**	**1**^**st**^	**2**^**nd**^	**3**^**rd**^	**Follow-up meeting**	**Final meeting**
Purpose	Initial recruitment		Setting action areas	Assessing change	
Number of PLWHA present	10	10	67	67	81

**Table 2:** Characteristics of the PLWHA included in the study (N = 67)

**Variable**	**N (%)**
**Marital status**	
Never married	**11 (15.7)**
Divorced	**24 (34.3)**
Cohabiting	**1 (1.5)**
Married	**9 (12.9)**
Widowed	**22 (31.4)**
**Employment**	
Never employed	**42 (63.0)**
Laid off work	**5 (7.5)**
Retired	**1 (1.5)**
**Education**	
None	**6 (8.6)**
Primary	**44 (62.9)**
Secondary	**16 (22.9)**
College	**1 (1.4)**
**Housing**	
Own	**7 (10.4)**
Rented	**53 (79.1)**
Friends	**1 (1.5)**
Parents	**5 (7.5)**
Street	**3 (4.5)**
Other	**1 (1.5)**
**Religion**	
Catholic	**47 (70.1)**
Protestant	**17 (25.4)**
Muslim	**2 (3.0)**
Other	**1 (1.5)**

### Non-compliance among PLWHA

Health workers as well as community members recognised the problems that PLWHA who use alcohol faced, but there were disagreements concerning priority areas. While health workers rated non-compliance with drugs highest, community members considered violence due to stress and legal problems as more important. Both recognised the risk alcohol posed with regard to unprotected sex. For PLWHA on ARVs, the timing of the clinics made it difficult for them to keep all their appointments. They also found the medication frequency inconvenient. Compliance was also poor because after taking medicines they felt hungry and weak and food was either too costly or not available. Whereas some of the latter issues also affected those who were not taking alcohol in that community, the participants felt that alcohol use among the PLWHA compounded the problems. From case studies and vignettes provided during the discussions both health workers and community members could identify the gross forms of alcohol intoxication and dependence, but the concept of alcohol misuse and hazardous drinking did not appear to be common or easily understood by community members. It also emerged that the PLWHA had not been screened for alcohol use at the health facilities and that the health workers did not routinely discuss issues of alcohol abuse. The patients felt that it was not easy to discuss alcohol related problems with the health workers. They felt that the social problems were not relevant medical problems that the health workers could help with. The health workers on their part were usually more concerned with the physical aspects of the illness. Thus there was poor communication between the clients and the health workers.

### Reasons given by the PLWHA for using alcohol

#### Stigma and for social acceptance

The PLWHA linked alcohol use to a desire to be accepted by the community and to prove that they are just like any other person. Some thought that using alcohol made them appear sexually attractive. Thus there was peer pressure to drink and alcohol was used to counteract the stigma associated with HIV and to gain confidence to discuss life issues freely.

#### To deal with psychological problems such as denial, hopelessness and revenge

Alcohol was seen to make one forget that they are HIV positive. Participants referred to the loss of hope, problems and worries that can be forgotten, temporarily, with alcohol use. Some also felt that PLWHA “drank intentionally to spread the virus”. They reasoned that the PLWHA were feeling hurt and wanted to spread the feeling to others as well. They used alcohol drinking to enhance social interaction entice others into sexual activities with the aim of spreading the viral infection.

#### Perceived “medicinal value” and physical addiction

People were ignorant of the dangers of using alcohol or thought that since alcohol is “strong” (concentrated) it could kill the HIV virus. It was also noted that ARV drugs made them feel hungry and since they had no food they resorted to alcohol use which numbed their feelings.

#### Poverty

**L**ack of money to buy food, easy availability of alcohol and drinking to get relief from the withdrawal symptoms of alcohol were the other reasons given for using alcohol. The participants were asked to identify important steps in reducing prevalence of harmful alcohol use in the community for PLWHA. Some of the PLWHA said that they sometimes engaged in commercial sex to cater for their basic needs. These factors, together with poor health, limited their economic opportunities and security. In this context, alcohol use, noted by PLWHA, community members and health workers to be prevalent in the community, was not only encouraged by poor living and social conditions, but also by cost (it is relatively cheap) and by the social pressure to use alcohol to escape the mental stress caused by poverty. This was exacerbated by social attitudes that did not discourage alcohol use, and misconceptions that in fact encouraged alcohol use, such as that alcohol could kill the HIV virus.

### Community resources

Through the use of spider diagrams and Venn diagrams, the participants identified a range of services in the community that could potentially address the problems that were identified. These included nutrition, psychosocial, primary health care (PHC), HIV prevention and treatment services, counselling, social, legal, information and referral support for PLWHA. However none of these dealt explicitly with the treatment of alcohol and drug related problems in the community or the needs of PLWHA on ARVs who use alcohol, and their adherence to treatment. Majengo clinic and the Comprehensive Care Clinics were run by medical doctors whereas Kariobangi and Comboni clinics were run by clinical officers and nurses. The research team leaders AO and MM accompanied by a community nurse visited the community and the clinics to verify the information obtained at the meeting. Discussions with the heads of the various clinics and the records kept revealed that alcohol related disorders were not commonly documented. Furthermore none of the health facilities used any screening instruments such as the AUDIT [24].

### Action areas

During the third meeting further discussions on the harmful effects of alcohol took place, exploring reasons why people drink and corrective measures that could be taken. The meetings were used to reinforce the steps people identified that they could take to reduce harmful drinking. Reflecting on the problems identified in the first two meetings, the participants noted that counselling and education were important. The community health nurses together with the authors organised education talks with the staff of the institutions earlier identified. These included the local health centres and the self-help groups. The health workers were taught how to use the AUDIT in identifying problem drinkers and how to recognise and manage alcohol related disorders such as withdrawal seizures. They were also advised to routinely ask questions on alcohol and compliance and to be more sensitive to the patients’ social needs. Other aspects on how to improve communication between the care providers and their clients were discussed. For the PLWHA basic information on the effects of alcohol on the body was provided.

The PLWHA were encouraged to form a registered group which could apply for funding on projects of their choice. The PLWHA and their family members were encouraged to support one another and to identify symptoms of harmful alcohol use among themselves. These were feasible within the network of PHC and community mental health resources in the community, although with additional support from the PRA research team. What was more difficult was implementing interventions aimed at improving the incomes of the affected PLWHA.

## Discussion

In our view the use of the PRA methods allowed us to explore the perceptions and feelings of PLWHA and those working with them in the community. Thus we were able to gain new insights into their problem. Furthermore the PRA approach allows the researchers to work together with those affected in finding solutions to their problem. For example, in this community there was clearly much misinformation about the physical effects of alcohol. The perception that strong alcoholic drinks had medicinal value and could kill the virus within the body may have led some PLWHA to drinking. We also found that the health workers and the patients were not communicating well. The clients to the going to the health centres did not feel free to talk about the social and economic aspects of their illness which was of more concern to them as opposed to the health workers who tended to concentrate more on the physical aspects of the illness. Such information could be useful in formulating interventions in minimising the risks associated with harmful alcohol use and noncompliance. Less easily addressed were the levels of economic and nutritional deprivation that led patients to engage in seemingly irrational behaviour that endangered their health. This calls for attention to the specific nutritional needs of PLWHA who use alcohol as part of their therapy, but also to the wider social and economic determinants that lead to harmful alcohol use. This calls for wider policies for economic and food security in vulnerable communities. Although social support for vulnerable groups is minimal in Kenya, perhaps direct financial support could be initiated for this group of patients.

In our experience the PRA methods need adequate time and patience if one is to draw useful information from the group works. This could be due to the fact that the participants were used to being passive observers in such situations. They were waiting to be told what to do rather than thinking how they could assist in the process. This was further compounded by cognitive impairment in some of the PLWHA. However it would be useful to explore the effectiveness and long term effects of this intervention. If proven successful then the methods could be integrated into PHC approaches to prevention and treatment of other illnesses as well.

### Limitations

The sample of PLWHA was obtained through snowballing so this may not be a representative sample from the community studied. However in this circumstance it was thought to be the best recruitment method considering the stigma that still prevails concerning HIV and alcohol use. Furthermore, the PLWHA who were recruited for the study were not objectively assessed for evidence of alcohol abuse. Since this was also mainly a qualitative study so we cannot be certain of the changes made nor attribute them solely to the PRA process. Nevertheless we hope that the findings from this study can form a basis for further quantitative studies.

## Conclusion

This action research highlights that wider chronic health and social problems in the community impede uptake of resources for prevention and treatment for HIV and AIDS, and that specific measures should be put in place, in collaboration with those affected, as part of health services and AIDS programmes.

## Competing interests

The author declared that they have no competing of interest.

## Authors’ contributions

CJO conceptualised the study and wrote it, AO and MM participated in the PRA process as facilitators. All authors read and approved the final manuscript.

## Pre-publication history

The pre-publication history for this paper can be accessed here:

http://www.biomedcentral.com/1471-2458/12/677/prepub
